# Different outcomes following parathyroidectomy in patients with uremic tumoral calcinosis: two case reports

**DOI:** 10.1186/s12882-023-03107-3

**Published:** 2023-03-15

**Authors:** Jialin Li, Xuan Li, Xiuhong Dong, Lin Ma, Zhentao Guo, Xuexun Chen

**Affiliations:** 1 Department of Nephrology, School of Clinical Medicine, Affiliated Hospital of Weifang Medical University, Weifang Medical University, Weifang, China; 2 Department of Gastroenterology, School of Clinical Medicine, Affiliated Hospital of Weifang Medical University, Weifang Medical University, Weifang, China

**Keywords:** Uremic tumor calcinosis, Parathyroidectomy, Treatment, Chronic renal failure, Hyperparathyroidism, Genetic testing, Case report

## Abstract

**Background:**

Uremic tumoral calcinosis (UTC) is a rare complication in hemodialysis patients, whose mechanism remains incompletely understood. We report two cases with UTC who experienced completely different patterns of regression following parathyroidectomy, although there were no significant differences in serum calcium levels, parathyroid hormone, or phosphorus production between the two patients.

**Case presentation:**

Case 1 had a substantial improvement in soft tissue calcification. However, in Case 2, one calcified mass was partially absorbed, while the others were aggravated with severe microvascular calcification and subcutaneous extravascular calcification. Whole-exome sequencing data revealed five mutation sites associated with atherosclerosis.

**Conclusion:**

The different outcomes in UTC patients after PTX are rare. Further studies are required to elucidate the mechanism of paradoxical changes occurring in patients with UTC after parathyroidectomy.

## Background

Metastatic calcification is a serious complication of end-stage renal disease (ESRD) in maintenance hemodialysis patients. It occurs most often secondary to hyperparathyroidism, where excessive vitamin D intake or severely damaged bone tissue results in elevated blood calcium. Calcium salts can deposit in many normal tissues throughout the body [1]. Following parathyroidectomy (PTX), many symptoms of renal osteoporosis can be relieved, associated biochemical markers can often be corrected, and calcified soft tissue is significantly resorbed [2]. However, bone renewal is reduced following PTX because of lower parathyroid hormone (PTH) levels, resulting in increased circulating calcium and promotion of calcification of vessels and soft tissues. Here, we report two cases of uremic tumoral calcification (UTC) following PTX with different outcomes. Case 1 showed improvement in clinical symptoms and imaging signs. Case 2 presented with severe microvascular calcification and subcutaneous extravascular calcification. Although PTX is an effective treatment for UTC, clinicians should be aware of this rare condition. To identify the underlying etiology of the different outcomes in the two patients, we performed whole-exome sequencing and identified three variant genes (*ATXN2L*, *POLR1B*, and *MMP19*) in Case 1 and two variant genes (*BMP4* and *TXNDC2*) in Case 2.

## Case presentation

### Case 1

**Fig. 1:**
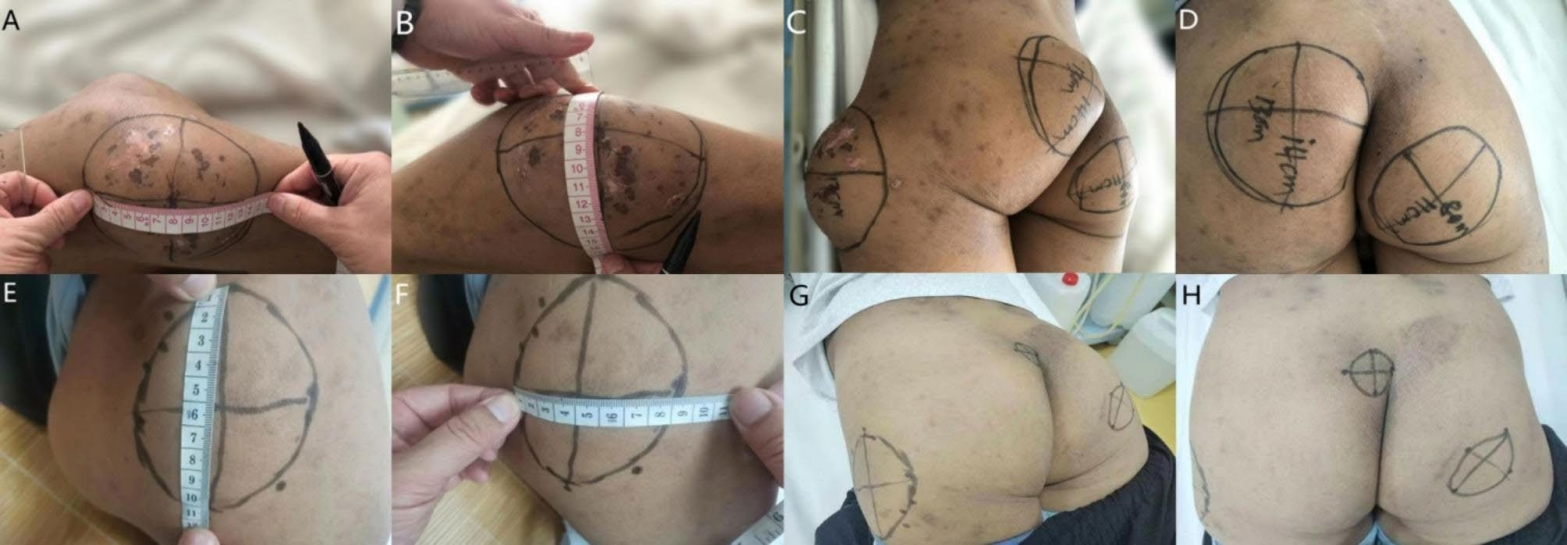
(**A**-**D**) Preoperative appearance of massive periarticular calcification with reduced joint mobility in case 1. (**E-H**) Significant reduction of the mass at 16 months after surgery in case 1

**Fig. 2:**
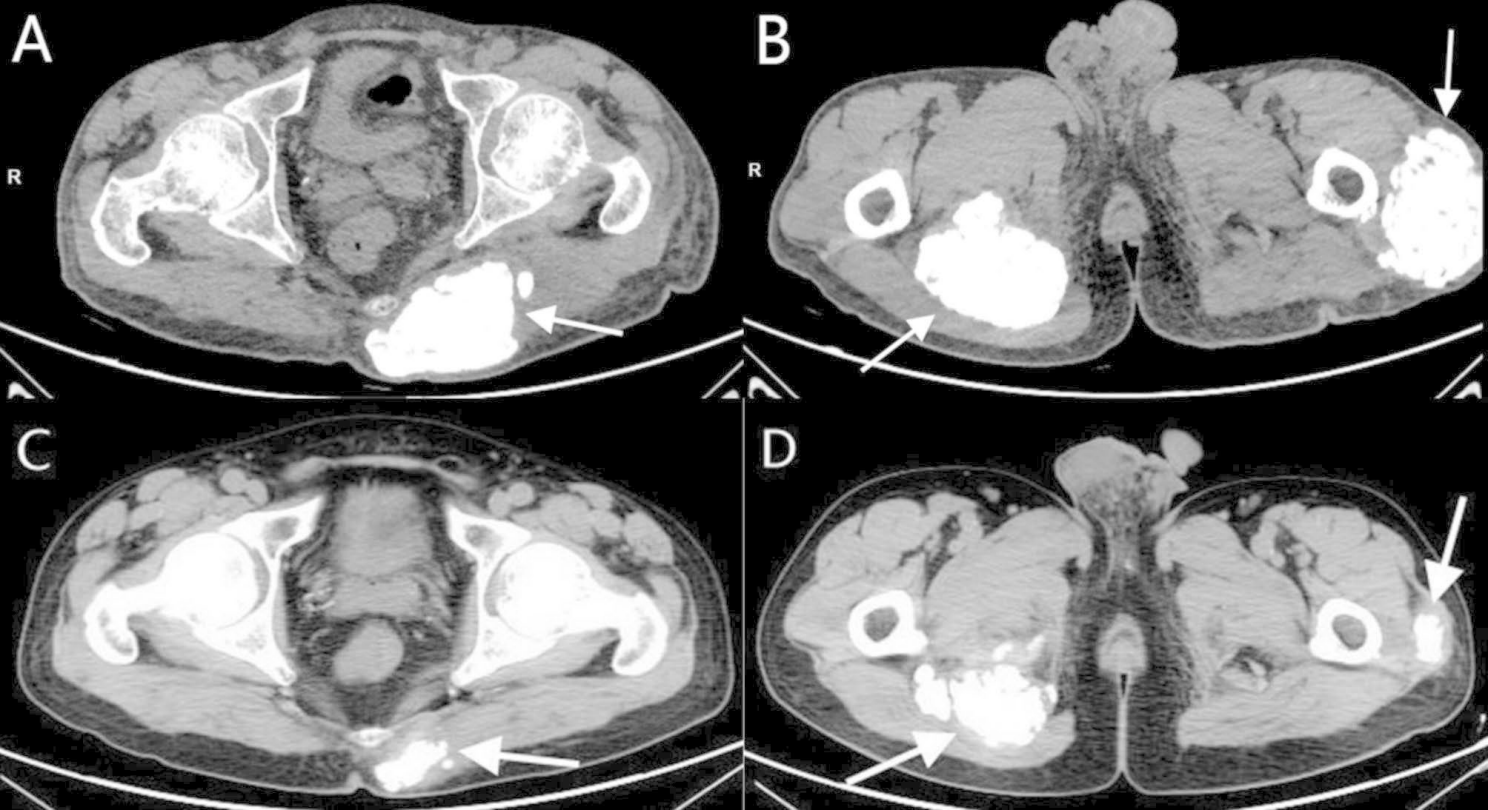
CT scan of the pelvis before (**A**, **B**) and after parathyroidectomy (**C**, **D**)in case 1

A 54-year-old male with history of hypertension had been on dialysis three times per week for 6 years because of ESRD. He presented with a 3-year history of whole-body itch followed by a 7-month history of hip pain with motion limitation and progressively enlarging masses on the hips. His blood pressure was 165/105 mmHg, and other vital signs were normal on admission. Physical examination revealed hyperpigmentation with multiple scratches and breaks. Three large, hard, fixed lumps were palpated on the lateral side of his left femur (15 × 17 cm), right hip (11 × 8 cm), and left hip (14 × 13 cm) (Fig. 1). Laboratory tests showed his serum PTH was 1384 pg/ml (reference range, 1–84 pg/ml). Computed tomography (CT) showed high-density shadows in the mitral valve area of the heart, aorta, and coronary arteries. Mass-like calcifications were present in the soft tissue of the left posterior side of the coccyx, the lateral side of the left greater trochanter, and the right ischium, with volumes of approximately 71.7 cm^2^, 117.9 cm^2^, and 132.9 cm^2^, respectively (Fig. 2). Neck ultrasound showed complex nodules with solid and cystic components near parathyroid area in both thyroid gland lobes (right lobe: 1.1 × 1.1 cm, 1.2 × 0.4 cm; left lobe: 1.8 × 1.1 cm, 1.4 × 0.7 cm). 99mTc-MIBI scintigraphy showed significantly increased radiation in the middle and lower part of left thyroid gland lobe at 15 min and 2 h. Diagnosis of secondary tumoral calcinosis associated with secondary hyperparathyroidism was made. A biopsy was not performed because of concern over poor healing. Later, in March 2021, he underwent total PTX with autograft (tPTX + AT). All parathyroid glands were identified and excised. And then the small part of gland (0.1 × 0.1 × 0.1 cm) implanted into the sternocleidomastoid muscle. Pathology showed nodular hyperplasia in all four parathyroid glands. Pathology showed nodular hyperplasia in all four parathyroid glands. His serum PTH level dropped to 166.2 pg/ml. Three days after the operation, the patient’s itching symptoms improved significantly, and the pain was relieved. During the 16-month follow-up, the masses on his left lateral femur and left and right hip shrank to 8.5 × 10 cm, 4 × 4 cm, and 5.5 × 5.5 cm, respectively (Fig. 1). CT scan showed that the mass volumes decreased to 37.9 cm^2^, 47.3 cm^2^, and 100 cm^2^, respectively (Fig. 2). His relevant clinical findings are shown in Table 1.

**Table 1:** Case 1: Laboratory values at various stages of the disease

DATE	Serum calcium (2.04-2.71mmol/l)	Serum Phosphate (1-1.6mmol/l)	Serum PTH level(15–65 pg/mL)	Ca x PO4	Clinical presentation
September 2020	2.56	2.17	882.5	5.56	Hyperparathyroidis
December 2020					Parathyroidectomy
December 2020	1.75	2.43	166.2	4.25	Post Parathyoidectomy
July 2021	2.46	2.24	18.1	5.51	Post Parathyoidectomy
September 2021	2.48	1.48	14	3.67	Post Parathyoidectomy
June 2022	2.2	2.12	70.48	4.66	Post Parathyoidectomy

## Case 2

**Fig. 3:**
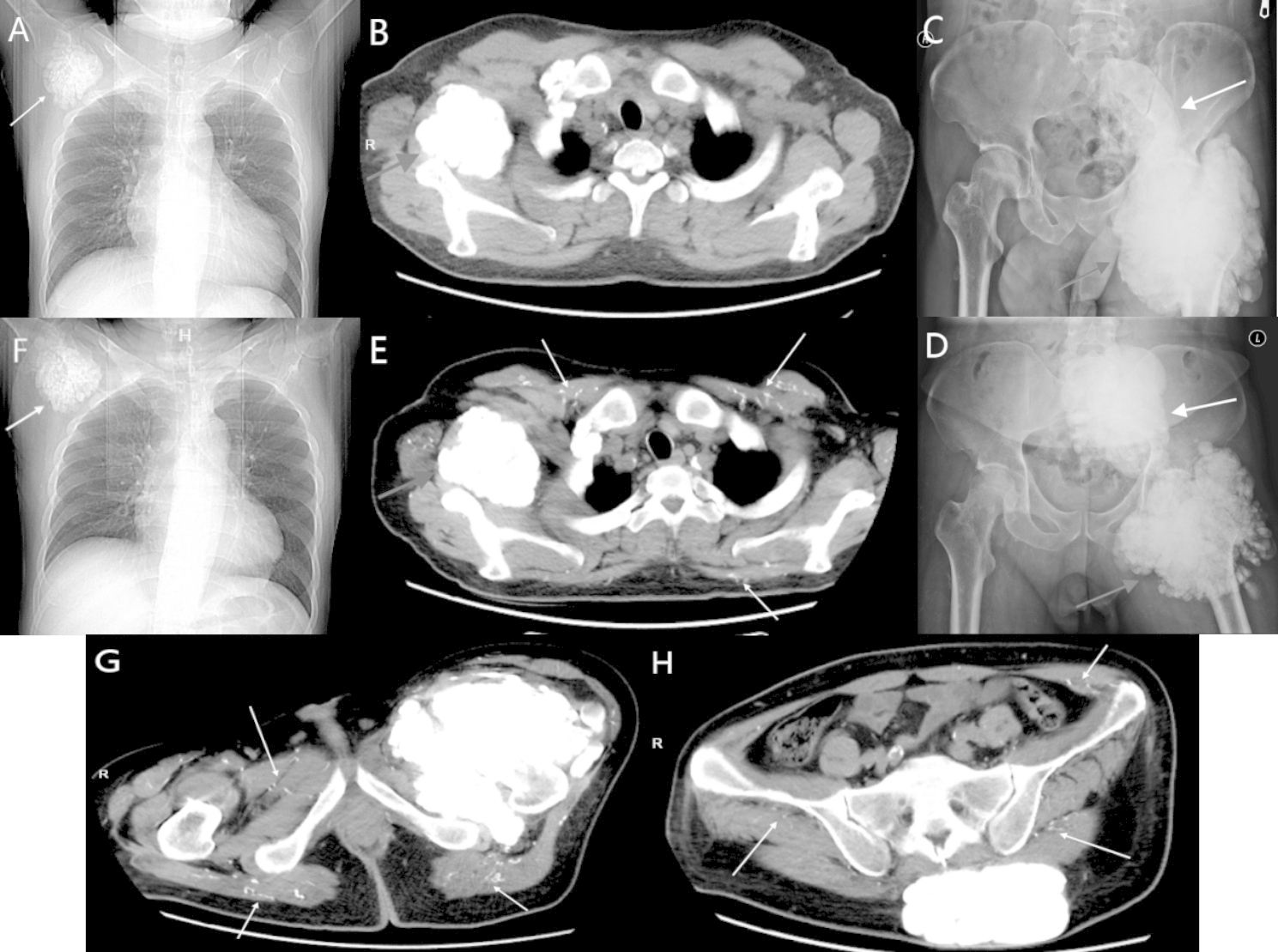
Standard X-ray shows the masses in the shoulder (**3 A, 3B**), the left hip joint (red arrow in **3 C**) and the pelvis (white arrow in **3 C**) before parathyroidectomy in case 2. Nineteen months after parathyroidectomy, the masses in the shoulder (**3E, 3 F**), the left hip joint (red arrow in **3D**), and the pelvis (white arrow in **3D**) in case 2. CT scan of shoulder joint calcification, shoulder joint (red arrow in **3E**) and vascular calcium (**3E, 3G-3 H**) 19 months after parathyroidectomy in case 2

In 2021, a 35-year-old male presented with left hip pain and whole-body itch for over 1 year. He had been attending hemodialysis sessions for approximately 7 years. He was administered the drug, mecobalamin (0.5 mg, three times per day), calcium carbonate (600 mg, twice per day), and calcitrio (0.25 µg, once per day) for five years. On admission, physical examination revealed a 20 × 15-cm mass on the left side of the pelvis and a 10 × 10-cm mass with a hard texture on the right shoulder. CT showed a large calcified mass of approximately 6 × 5 cm in the right shoulder (Fig. 3A and B). Plain radiograph showed a multilobar calcified mass near the left hip joint, with a volume of approximately 19 × 17 cm (Fig. 3C). 99mTc-MIBI parathyroid scintigraphy was normal. Laboratory results were as follows: serum creatinine: 828 µmol/L, serum calcium: 2.46 mmol/L (reference range, 2.04–2.71 mmol/L), serum phosphorus: 2.85 mmol/L (reference range, 1–1.16 mmol/L), serum alkaline phosphatase: 120 U/L (reference range, 45–125 U/L), and serum PTH: 650.3 pg/ml (reference range, 15–65 pg/ml). The patient underwent tPTX + AT. The gland was implanted into the sternocleidomastoid muscle using the same method. Pathology showed nodular hyperplasia in all parathyroid glands. After the operation, his PTH dropped to 35.51 pg/ml, blood calcium was 1.82 mmol/L, and he initially received calcitriol (0.25 µg, twice per day) and calcium carbonate (600 mg, twice per day), which were adjusted according to laboratory results. The patient’s hip pain was relieved rapidly following surgery. Nineteen months later, the patient was readmitted because of gastrointestinal bleeding. Relevant follow-up data are shown in Table 2. CT revealed that the mass near the left hip joint shrunk from 19 × 17 cm (Fig. 3C) to 14 × 13 cm (Fig. 3D). However, there was no significant change in the right shoulder mass (Fig. 3E F). In contrast, the calcified mass in the pelvis increased in size (Fig. 3D). Additionally, the patient developed apparent microvascular calcifications and subcutaneous extravascular calcifications as shown by CT (Fig. 3E and G H).

**Table 2:** Case 2: Laboratory values at various stages of the disease

DATE	Serum calcium (2.04-2.71mmol/l)	Serum Phosphate (1-1.6mmol/l)	Serum PTH level(15–65 pg/mL)	Ca x PO4	Clinical presentation
December 2020	2.5	2.42	461.4	6.05	Hyperparathyroidis
March 2021	2.46	2.85	650.3	7.01	Hyperparathyroidis
March 2021					Parathyroidectomy
March 2021	1.82	2.66	35.51	4.84	Post Parathyoidectomy
December 2021	2.11	2.24	18.1	4.73	Post Parathyoidectomy
February 2022	2.02	2.9	43.8	5.86	Post Parathyoidectomy
September 2022	2.14	2.29	55.5	4.90	Calciphylaxis

## Whole-exome sequencing

It was a very rare situation that uremic tumoral calcification happened different outcomes after PTX. To identify the underlying etiology of the different outcomes, we performed whole-exome sequencing, which was permitted by patients and approved by the Ethics Review Committee of Affiliated Hospital of Weifang Medical University. Sequencing data were compared using the Illumina DRAGED Bio-IT Platform and the mutation sites were filtered. Five valid candidate genes were obtained based on the gene enrichment classification of the Kyoto Encyclopedia of Genes and Genomes[3–5], and the relative gene sets of Lipid_atherosclerosis and Fluid_shear_stress_atherosclerosis were selected. Three coding variants associated with atherosclerosis with genome-wide significance were identified in Case 1: rs28846866 in *ATXN2L*, rs113300001 in *POLR1B*, and rs56236602 in *MMP19*. Two reference single-nucleotide polymorphism variants (rs54417522 in *BMP4*, rs9887388 in *TXNDC2*) reported to be associated with atherosclerosis were found in Case 2.

## Discussion and conclusions

UTC is a rare disease characterized by soft tissue calcification around joints in uremic patients. It always presents with swelling, pain, and limited motion in multiple or individual joints [6, 7]. The internal mechanisms of UTC remain unclear. Previous studies reported that elevated calcium phosphate production is most closely associated with soft tissue calcification [8, 9]. Other risk factors include hyperparathyroidism, hyperphosphatemia, calcium-containing phosphate binders, and inappropriate use of active vitamin D. Metabolic alkalosis and tissue damage may also play a role in soft tissue calcification [10, 11]. Medical treatment includes dietary phosphorus restriction, calcium-free phosphate binders, and frequent dialysis with low-calcium dialysis solutions. However, these treatments are usually difficult to implement and ineffective [1, 12, 13].

PTX is effective for UTC patients with secondary hyperparathyroidism [1]. Some studies showed that UTC was lessened or disappeared with improved systemic symptoms following PTX [14–16]. Resorption of UTC may be associated with the sudden drop in PTH level, and subsequent decrease of bone resorption and increase of bone formation [17, 18]. In Case 1, the symptoms were relieved significantly after PTX. However, there were paradoxical changes in Case 2. UTC was lessened near the left hip joint, there was no significant change of UTC in the shoulder, and there was an increase of UTC in the pelvis. CT confirmed that he unfortunately developed severe microvascular calcification and subcutaneous extravascular calcification.

The simultaneous paradoxical changes that occurred in Case 2 have rarely been reported. London et al. concluded that the degree of vascular calcification in ESRD patients is associated with lower bone activity and dynamic bone disease. Reduced bone remodeling is significantly associated with aging and reduced parathyroid “activity” [19]. Following PTX, the bone turnover rate decreases from a state of high conversion. Consequently, calcium uptake by bone is reduced. Excess calcium phosphate is then deposited in the vascular walls of soft tissues, leading to severe microvascular calcification and subcutaneous extravascular calcification [20, 21].

Postoperatively, we applied calcium carbonate and osteotriol to avoid and treat hypocalcemia, which may also contribute to calcification. However, Case 1 did not develop microvascular calcification, although he also had hyperphosphatemia and elevated calcium phosphate production, similar to Case 2. To identify the underlying etiology of the different outcomes of the two patients, we performed whole-exome sequencing in both patients. The three variant genes in Case 1 were *ATXN2L*, *POLR1B*, and *MMP19*, which were different from the variants in Case 2, *BMP4* and *TXNDC2*. Early development of vascular calcification is associated with overexpression of BMP4 in CKD [22]. Additionally, increased BMP4 in serum was strongly correlated with aortic calcium levels, suggesting that it could be used as a biomarker of calcification associated with CKD [23]. The present study raised an interesting question as to why Case 2 did not develop microvascular calcification before PTX. We hypothesize that the BMP4 mutant gene is repressed at high PTH levels. After PTX, the BMP4 mutant gene is activated, blocking the uptake of UTC and leading to excess calcium phosphate deposition in the vessel wall. Presently, the associations between the other genes and calcification in CKD remain unclear. Further studies are needed to confirm their specific effects in CKD.

In conclusion, the different outcomes in UTC patients following PTX are rare. For the first time, we performed whole-exome sequencing and identified five potential candidate genes. Paradoxical changes occurring in the same patient following PTX have not been reported previously. Further studies are required to elucidate the mechanism.

## Data Availability

The datasets generated and analysed during the current study are available in the NCBI repository,PRJNA910968.

## Abbreviations

ESRD: end-stage renal disease
UTC: Uremic tumoral calcinosis
PTX: parathyroidectomy
PTH: parathyroid hormone
CT: Computed tomography.
